# Imaging Bacteria with Radiolabelled Probes: Is It Feasible?

**DOI:** 10.3390/jcm9082372

**Published:** 2020-07-25

**Authors:** Alberto Signore, Vera Artiko, Martina Conserva, Guillermina Ferro-Flores, Mick M. Welling, Sanjay K. Jain, Søren Hess, Mike Sathekge

**Affiliations:** 1 Nuclear Medicine Unit, Department of Medical-Surgical Sciences and of Translational Medicine, Faculty of Medicine and Psychology, Sapienza University of Rome, 00189 Rome, Italy; martina.conserva977@gmail.com; 2 Center for Nuclear Medicine, Clinical Center of Serbia, Faculty of Medicine, University of Belgrade, 101801 Beograd, Serbia; vera.artiko@gmail.com; 3 Department of Radioactive Materials, Instituto Nacional de Investigaciones Nucleares, Carretera Mexico-Toluca S/N, La Marquesa, Ocoyoacac 52750, Estado de Mexico, Mexico; guillermina.ferro@inin.gob.mx; 4 Interventional Molecular Imaging Laboratory, Department of Radiology, Leiden University Medical Center, 2333 ZA Leiden, The Netherlands; m.m.welling@lumc.nl; 5 Center for Infection and Inflammation Imaging Research, Johns Hopkins University School of Medicine, Baltimore, MD 21205, USA; sjain5@jhmi.edu; 6 Department of Radiology and Nuclear Medicine, Hospital South West Jutland, University Hospital of Southern Denmark, 6700 Esbjerg, Denmark; soren.hess@rsyd.dk; 7 Nuclear Medicine Department, University of Pretoria, Pretoria 0001, South Africa; Mike.Sathekge@up.ac.za

**Keywords:** infection, bacteria, radiopharmaceutical, molecular imaging, nuclear medicine

## Abstract

Bacterial infections are the main cause of patient morbidity and mortality worldwide. Diagnosis can be difficult and delayed as well as the identification of the etiological pathogen, necessary for a tailored antibiotic therapy. Several non-invasive diagnostic procedures are available, all with pros and cons. Molecular nuclear medicine has highly contributed in this field by proposing several different radiopharmaceuticals (antimicrobial peptides, leukocytes, cytokines, antibiotics, sugars, etc.) but none proved to be highly specific for bacteria, although many agents in development look promising. Indeed, factors including the number and strain of bacteria, the infection site, and the host condition, may affect the specificity of the tested radiopharmaceuticals. At the Third European Congress on Infection/Inflammation Imaging, a round table discussion was dedicated to debate the pros and cons of different radiopharmaceuticals for imaging bacteria with the final goal to find a consensus on the most relevant research steps that should be fulfilled when testing a new probe, based on experience and cumulative published evidence.

## 1. Introduction

The diagnosis of bacterial infections remains a serious medical challenge, as they are among the main causes of mortality and morbidity worldwide. Nuclear medicine lacks specific radiopharmaceuticals to discriminate infection from sterile inflammation, and radiology often has poor sensitivity in detecting infective foci, especially in the early phases or in deeply seated infections. The diagnosis of infection often relies on serological markers and clinical symptoms, the gold standard being the isolation of the pathogen [1,2].

Indeed, radiological imaging modalities, such as X-rays, ultrasound (US), computed tomography (CT) and magnetic resonance imaging (MRI) provide an indication of the anatomical area of lesion only after the formation of a morphological alteration.

For Nuclear medicine imaging, many radiopharmaceuticals have been synthetized to detect physiological and biochemical changes at the early stages of infection, but to date, none have been made commercially available. Appropriate radiopharmaceuticals should enable early diagnostic imaging, identifying the pathogen and its biological characteristics, thus monitoring the therapy response as well as identifying drug-resistant strains, and the prognosis. The ideal one should have fast accumulation, high retention at the site of infection with fast clearance from non-infected tissues, with low absorbed radiation dose. In addition, it must be readily available, with simple labelling, inexpensive, repeatable and safe [3].

**Figure 1:**
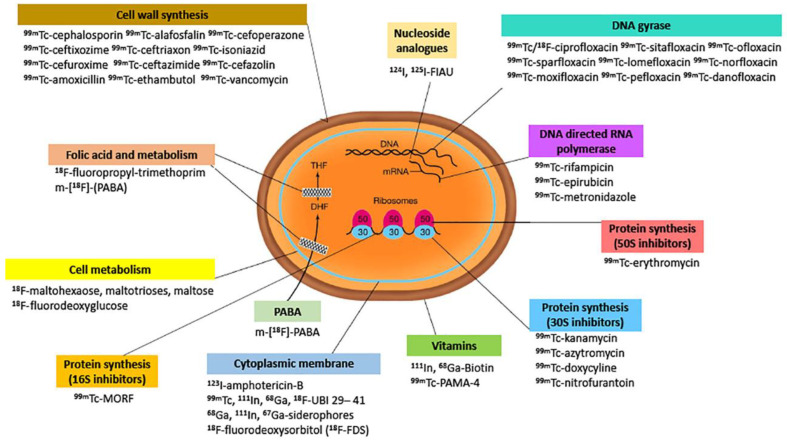
Schematic representation of most radiopharmaceuticals proposed for targeting bacteria, according to their mechanism of action. However, none are able, in humans, to differentiate between infection and inflammation with high diagnostic accuracy (>95%).

Nowadays, according to these criteria, the detection of infection by non-specific radiopharmaceuticals could be performed with metabolism-based particles (nucleoside analogues, sugars, cell wall components, components based in iron metabolism), antimicrobial peptides, antibiotics (fluoroquinolones, cephalosporins, antifolates), immunoglobulins and cytokines labelled with gamma- or positron-emitting isotopes (^18^F, ^64^Cu, ^68^Ga, ^99m^Tc, ^111^In, ^67^Ga etc.), aptamers/oligomers, bacteriophages, and vitamins (Figure 1), [4,5,6,7]. However, each approach has its limitations and investigations lack standardization.

It is now well known that [^18^F]-fluorodeoxyglucose ([^18^F]FDG) is taken up by the cells involved in the inflammatory response (e.g., neutrophils, macrophages and activated leukocytes) because they express high levels of glucose transporters like malignant cells (albeit not to the same extent), and in addition, circulating cytokines seem to increase the affinity of these glucose transporters for [^18^F]FDG [8] and it has recently been shown that some bacterial strain can also bind [^18^F]FDG [9].

Nonetheless, the non-specific nature of [^18^F]FDG may also be a hindrance in other settings, as the distinction between aseptic inflammation and infectious foci is difficult. Much work has been invested in optimizing the use of [^18^F]FDG.

On the other hand, in recent years, some more specific radiopharmaceuticals were developed in different nuclear medicine fields, such as the oncological one (e.g., prostate-specific membrane antigen (PSMA)-imaging for prostate cancer and various radiopharmaceuticals for neuroendocrine tumours). Just as tumour imaging, more specific radiopharmaceuticals are also being investigated for infection, even if ^99m^Tc and ^111^In-labelled white-blood-cells (WBC) remain the gold standard technique for the nuclear medicine imaging of infections [10].

Possible reasons for not having a commercially available radiopharmaceutical yet for imaging bacteria, could be the high costwith respect to the available market, but more likely, the lack of reproducibility of the published data. This is because different animal models are often used, different bacteria, different methods of image acquisition and interpretation, different quality controls on radiopharmaceuticals, etc.

Based on the active discussion during the round table session on “Bacteria imaging” at the Third European Congress on Infection/Inflammation Imaging, we aimed with this paper to generate a consensus document on the minimum requirements needed for more infection-specific radiopharmaceuticals for bacteria imaging (i.e., radiopharmaceuticals aimed directly at the microorganism and not only at the secondary inflammatory response), looking also at the upcoming technologies that could improve diagnosis and patient comfort, especially in areas not readily accessible for sampling or biopsies.

### The Role of Pathogenic Bacteria in Infections and Bacteria-Specific Features for Targeting

Planktonic bacteria are free-living bacteria, which are generally treatable with antibiotics, but when they adhere to a surface, develop a biofilm.

Bacterial biofilms are groups of bacteria that are embedded in a self-produced matrix of extracellular polymeric substances (EPS), adhering to each other and usually to a surface, thus, allowing intense interactions to occur, including cell–cell communication, altered phenotypes with respect to growth rate and gene transcription [11]. Biofilm-embedded bacteria represent a serious clinical problem in medicine, because their infections are notoriously difficult to treat due to extreme resistance to antibiotics.

Antibiotics are drugs of natural or synthetic origin that can kill (bactericidal drugs) or inhibit (bacteriostatic drugs) cell growth. Most bactericidal antimicrobials are: cephalosporins, carbapenems, glycopeptides, fluoroquinolones, polymyxins that inhibit DNA synthesis, RNA synthesis, cell wall synthesis, or bacterial protein synthesis.

Fluoroquinolones (FQs) are bactericidal antibiotics effective for both Gram-negative and Gram-positive bacteria, and ciprofloxacin is the most widely used antimicrobial agent among FQs. The action of ciprofloxacin results from the inhibition of the enzymes topoisomerase II (DNA gyrase, gyrA and B) and topoisomerase IV (grlA and B), which are required for bacterial DNA replication, transcription, repair, strand super coiling repair, and recombination. Resistance to FQs in bacteria is mainly mediated by alterations in DNA gyrase and topoisomerase IV with specific amino acid substitutions in the “quinolone-resistance determining region” (QRDR) in gyrA and B subunits of DNA gyrase and parC and parE subunits of topoisomerase IV. Other common mechanisms are the reduced permeability/increased efflux of ciprofloxacin across bacterial membranes, and plasmids that protect cells from the lethal effects of FQs [12,13].

Toxic effects of FQs on humans have been attributed to their interactions with different receptor complexes, such as the blockade of the GABAa receptor complex within the central nervous system, leading to excitotoxic type effects and oxidative stress [14]. These toxic effects, however, are unlikely to be noted at a tracer dose that is used for PET/SPECT imaging because of the relatively high IC50 of FQs with respect to the micromolar quantities injected as radiopharmaceuticals.

Cephalosporins are one of the largest families of β-lactam antibiotics. They are bactericidal agents and have the same mode of action as other beta-lactam antibiotics (such as penicillin). Cephalosporins disrupt the synthesis of the peptidoglycan layer of bacterial cell walls by binding to penicillin-binding proteins (PBPs), causing the walls to break down and eventually the bacteria die. The three fundamental mechanisms of antimicrobial resistance are: the enzymatic degradation of antibacterial drugs, changes in PBPs, and changes in membrane permeability to antibiotics. The most important mechanism of resistance to cephalosporins is the destruction of beta-lactam rings by β-lactamase enzymes. Mutational changes in original PBPs or the acquisition of different PBPs will lead to the inability of the antibiotic to bind to the PBPs and inhibit cell wall synthesis. A change in the number or function of the general diffusion porin channels can reduce the permeability.

Since antimicrobial compounds act on processes that are unique to bacteria, it has been proposed that radiolabelled antibiotics should be able to distinguish microbial from non-microbial inflammation, because of their specific binding to the causative agents.

Another important problem of antibiotics is the risk of a resistance mechanism in bacteria that are increasingly common and could prevent the specific binding of the antibiotic ligand, leading to poor uptake. Furthermore, since antibiotics are designed to kill or disable the bacteria with high potency, many radiolabelled antibiotics do not accumulate in the bacteria, and thus may not provide a high enough contrast from the surrounding mammalian cells [2]. For these reasons the gold standard for bacterial infection imaging has not yet been found. Further in understanding the pathogenesis of infectious diseases goes beyond identifying the site of infection and disease-causing pathogen. Infectious lesions are characterized by a heterogeneous microenvironment which may include spatial physical and chemical differences as well as varied immune responses. These non-specific radiotracers targeted at these microenvironment biomarkers may provide valuable information regarding the heterogeneity of infection sites and have the potential to inform on the efficacy of antimicrobial treatments [14,15] as well as host-directed therapies [13,16,17]. Hopefully in the future we will have many radiopharmaceuticals available, tailored for specific pathogens, and clinical conditions, thus having the maximum specificity (see Table 1).

## 2. Imaging Bacteria in Animal Models

Nuclear medicine imaging improved the diagnosis of infections through the development of several radiopharmaceuticals that are constituted by different molecules such as “antibodies or fragments, antibiotics, antimicrobial peptides, bacteriophages”, but none of these are really “infection specific”. The main limits include a low specificity, low bacterial mass, unclear mechanisms of action, the presence of biofilm that limits their penetration and the host immune response. Moreover, the location of the bacterial target influences the choice of radiopharmaceuticals and its development [18,19,20,21].

For this purpose, for imaging infections, several steps should be followed to develop an efficient radiopharmaceutical to target bacteria. Firstly, test the specificity through in vitro binding assays and, secondly, evaluate the specificity in vivo choosing the best animal model. Then, translate the preclinical results to humans.

The Teflon cage model is an example of a standardized reproducible model to study bacterial infections in animals. In this model, a Teflon cage is implanted into the back of the mouse under the skin, by easy surgical procedure with a small incision of 5 mm. Despite that this model requires surgical intervention, it provides several advantages such as: the possibility to locally inject a known number of bacteria; the possibility to accurately evaluate the bacterial mass; the possibility to withdraw samples of fluids or cage to measure the bacteria and radiopharmaceutical concentration over time; the possibility to study biofilm formation [22].

The following radiopharmaceuticals might be considered the progenitors for bacterial infections by SPECT imaging because there are more data both in animals and in humans: 99mTc/18F-UBI 29-41, 111In-biotin [23,24]. Ubiquicidin (UBI) is a cationic human antimicrobial peptide fragment. However, radiolabelled UBI 29-41 is not widely used as a clinical agent due to the lack of a commercial kit approved worldwide for human studies (only available in Mexico), however, there are over 30 clinical studies performed underlying its usefulness in imaging infections [25,26]. Radiolabelled ciprofloxacin, after promising results in animals, when tested in humans, showed very discordant results in terms of specificity and sensibility [24]. In the majority of papers on Gram-negative bacteria, promising results were reported by using radiolabelled sugars (glucose, sorbitol, maltose, maltohexaose and ^18^F-fluoromaltotriose and ^18^F-fluoroacetamido-d-glucopyranose (FAG)) [27]. Indeed, several groups showed a high specificity of [^18^F]FDS binding to *E. coli* or *K. pneumoniae* [28] in animals. Several other studies have demonstrated similar results, but always in animal models [29,30]. In addition, other sugars such as ^18^F-fluoromaltohexaose (FMH) [31], 6″-^18^F-fluoromaltotriose and ^18^F-fluoroacetamido-d-glucopyranose (FAG), [32] were revealed to be sensitive and specific radiopharmaceuticals for the detection of *E. coli* [33]. In addition, a new Gram-negative bacterial infection-specific radiopharmaceutical has been developed: ^99m^Tc-polymyxin B. The polymyxin B is an antibiotic, usually used for multidrug-resistant Gram-negative bacteria, that acts like an amphipathic antimicrobial peptide. Similarly, D-amino acids, molecules targeting the folate pathway in bacteria and siderophores have also been studied as bacterial specific imaging agents [34,35,36,37,38,39].

In conclusion, the results highlighted the availability of many promising PET radiopharmaceuticals for bacterial imaging, even if imaging bacteria is still a difficult and challenging task. Animal models should be carefully selected and standardized, as well as bacteria strains. Experimental design should include in vitro and in vivo studies with appropriate controls and details of *S. aureus*, injected dose and bacterial number. A consensus document about how to test (in vitro and in animals) new bacterial imaging agents may allow a standardization of procedures and a better comparison between different agents.

There is a lack of knowledge whether it is possible to develop an all-purpose radiopharmaceutical to image all bacterial strains. Nowadays, this remains an open goal, difficult to achieve, however, at the same time, crucial for the management, treatment and follow-up of patients with suspected bacterial infections.

## 3. Imaging Bacteria in Humans

Despite excellent pre-clinical studies, radiopharmaceuticals for imaging bacteria in humans are still under development [40]. An unmet need, therefore, remains in the clinical differentiation of inflammation from infection. Bacterial-specific imaging is a viable attempt to cater for this need, and efforts in this regard must be encouraged, especially given the significant morbidity and mortality burden that infections continue to cause.

In humans, following the target-based classification, the best radiopharmaceuticals for bacteria imaging are: pathogen-specific tracers, antimicrobial tracers and microenvironment tracers.

In particular, tracers with the highest translational potential are antimicrobial peptides such as UBI 29-41, bacterial carbohydrates, nucleoside/thymidine analogue, folic acid, siderophores and antibiotics such as Trimethoprim and Vancomycin.

Antimicrobial peptides have been successfully radiolabelled and tested for infection imaging in animal models and humans. The first radiolabelled antimicrobial agent evaluated for human use was ^99m^Tc-ciprofloxacin [41]. Disappointing results from its application in humans led to its withdrawal from the market. In particular, its specificity and sensitivity for infections were questioned in several studies, probably due to the formation of several radiolabelled chemical species with different biodistribution profiles [42,43,44]. Imaging time-points were also questioned, up to 4 h in one study and up to 24 h in another. Many other antibiotics, including fluoroquinolones, cephalosporins, and anti-tuberculosis drugs, have since been successfully labelled with a suitable radionuclide and tested in preclinical studies [45].

A radiolabelled antimicrobial peptide that has gained popularity in the clinic is radiolabelled ubiquicidin, a human antimicrobial peptide present in the respiratory epithelium. Its fragments have been successfully labelled with ^68^Ga for PET imaging and ^99m^Tc for SPECT imaging [46]. The basis for the use of the fragment UBI 29-41 is its ability to be attracted to the negatively charged bacterial cell wall, itself being positively charged. ^99m^Tc-UBI 29-41 scintigraphy has an excellent diagnostic performance in the evaluation of musculoskeletal infection. The addition of CT morphologic imaging to planar and SPECT-only imaging led to an increase in diagnostic performance and an improvement in diagnostic confidence in differentiating soft tissue from bone infection, as well as a higher inter-observer agreement [26].

Moreover, gallium-68-based infection-imaging agents are in demand to detect infection foci with high spatial resolution and sensitivity. ^68^Ga-NOTA-UBI 29-41 is an efficient and sensitive radiopharmaceutical of the in vivo imaging of infection and has exhibited significant uptake ratios between muscular infection and inflammation [47]. Further clinical evaluation of this novel metabolic tracer is warranted to investigate its potential use as a first-line PET/CT infection-imaging agent. ^68^Ga-UBI prepared using the NOTA-UBI kit is a potential agent in targeting infections associated with disease conditions including diabetic foot, cellulitis and fracture. Indeed, biodistribution studies with ^68^Ga-NOTA-UBI 31-38 revealed a specific uptake of the complex in infected muscle, compared to inflamed muscle. This was the first report on ^68^Ga labelled NOTA-UBI 31-38 fragment for prospective infection imaging [48].

Furthermore, ^18^F-fluorodeoxysorbitol has been successfully synthesized from ^18^F-FDG, and it showed specific uptake in the cultures of *E. coli* and *K. pneumoniae*. No uptake of ^18^F-fluorodeoxysorbitol was seen in Gram-positive organisms, normal human cells, or cancer cells. The probe was able to differentiate the infection due to Enterobacteriaceae from sterile inflammation, and the PET signal disappeared after successful treatment [28].

Antibiotics such as ^99m^Tc-vancomycin and ^18^F-fluoropropyl-trimethoprim target peptidoglycan precursors on bacterial (Gram-positive bacteria) membrane and inhibit the bacterial cell wall synthesis [49]. Although they are bacteria-specific and -targeting drug-resistant Gram-positive bacteria, biodistribution studies revealed a high liver uptake, high background activity and low sensitivity. Therefore, they are not used for routine clinical application yet.

Fialuridine is a nucleoside analogue that is a substrate for the bacterial thymidine kinase enzyme but is not acted on by the human form of the enzyme. This is its basis for use as a potential molecular probe for infection imaging. However, ^124^I-FIAU lacks specificity in patients with prosthetic joint infections, and it has a high background signal in uninfected muscle, presumably due to host mitochondrial metabolism [50].

## 4. Conclusions

To conclude, the metabolic imaging of infection holds great promise. The focus of its application is shifting from mere diagnosis of infection to prognostication, to predict the response to treatment, to identify resistant strains and to identify and target at-risk patients for prevention.

New possibilities emerge also by the application of dual-isotope imaging after the simultaneous administration of two radiopharmaceuticals or one radiolabelled and one fluorescent or one paramagnetic.

It is hoped that when PET/MRI and SPECT/MRI achieve greater clinical utility, these hybrid systems may have even more applications in infection imaging due to the high sensitivity of MRI for soft tissues and oedema. It is also hoped that hybrid molecular probes for multimodality imaging soon may gain clinical relevance for infection imaging. Focused research is pointing toward a time when molecular probes will be able not only to detect infection but also to identify the offending organism and its biologic characteristics [47].

Overall, this article highlights that standardized protocols and consensus guidelines regarding animal models of infection are needed, preferably written by a joint technical committee. The optimization of preclinical research should be directed in improving the sensitivity for a broader range of microbes rather than species-specific probes. This broader range approach, in combination with the growing opportunities for imaging the microenvironment at infection sites, may help to resolve the challenges in the development of the radiopharmaceuticals that can differentiate sterile inflammation from infection, and thus, making the imaging of bacteria a viable option for future clinical studies.

## Figures and Tables

**Figure 2:**
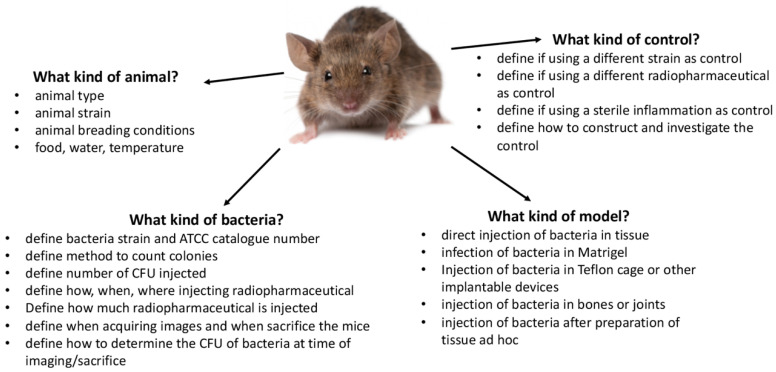
Schematic representation of the most relevant aspects to be taken in consideration when planning new experiments in animal models for targeting bacteria.

**Table 1:** Aspects to be considered for the improvement of bacteria imaging.

**Pathogen-Specific Radiopharmaceuticals**
✓ Sensitivity for a broader range of microbes rather than species-specific probes ✓ Screen potential radiopharmaceuticals in whole bacterial cell ✓ Always use referenced bacterial strains and specify Colony Forming Units (CFUs)
**Antimicrobial Radiopharmaceuticals**
✓ Library of antibiotics—very high affinity to targets (accumulation and slow clearance) ✓ Radiochemistry to balance T1/2 of the radioisotope and the parent drug
**Vitamins and Sugars**
✓ Define the metabolic role and pathway of new radiopharmaceuticals derived from vitamin’s or sugar’s analogues ✓ Test specificity in different bacteria strain and binding to eukaryotic cells
**Optimize Labelling Protocols and Quality Controls**
✓ Consensus guidelines on minimal required in vitro quality controls to better characterize the new radiopharmaceuticals (labelling efficiency, specific activity, mass spectroscopy, chromatography data, radiopharmaceutical stability in saline and plasma, etc.) ✓ Determine the Kd for tracer target specificity ✓ Test on living bacteria in vitro (binding at 37 °C and 4 °C, binding to living and killed bacteria, competitive binding assay, etc.)
**Optimize Animal Models (Figure 2** **)**
✓ Standardized protocols and consensus guidelines regarding animal models of infection are needed ✓ Trials with new probes compared with commonly used radiopharmaceuticals in clinical settings and other modalities (e.g., fluorescence imaging) ✓ Include positive and negative control tracers like D,L analogues or scrambled peptides, etc. ✓ Consider competition studies ✓ Always provide information about the model (injected CFUs, time of imaging and sacrifice, CFU recruited from infected site at different time points, etc.) ✓ Provide information on the animal used (strain, culture, food, drinking water, age, sex, body weight, etc.)
